# Oocyte retrieval in social fertility preservation: challenging traditional beliefs on gonadotropin dosing and follicular size

**DOI:** 10.1007/s10815-024-03138-1

**Published:** 2024-05-20

**Authors:** Einat Haikin Herzberger, Mor Semo, Kim Soifer, Netanella Miller, Nitzan Goren Gepstein, Roni Rahav, Mattan Levi, Amir Wiser

**Affiliations:** 1 IVF Unit, Department of Obstetrics and Gynecology, Meir Medical Center, 59 Tchernichovsky St., 44281 Kfar Saba, Israel; 2 Faculty of Medicine, Tel Aviv University, Tel Aviv, Israel

**Keywords:** Ovarian stimulation, Oocyte cryopreservation, Elective fertility preservation, Social fertility treatment

## Abstract

**Objectives:**

To investigate treatment approaches for fertility preservation patients, with a focus on timing of oocyte retrieval, and to determine whether their characteristics differ from those of other IVF patients. Additionally, to evaluate the significance of follicle size on triggering day in the context of fertility preservation.

**Methods:**

This retrospective cohort study was conducted in a tertiary, university-affiliated medical center. It compared 140 matched patients undergoing social fertility preservation to 140 patients undergoing IVF treatment due to male factor infertility.

**Results:**

Patients undergoing fertility preservation received a higher initial gonadotropin dose and had more oocytes retrieved than the control group. Within the fertility preservation cohort, a negative correlation was observed between the rate of large follicles and the number of retrieved oocytes. While there was no significant association between rate of large follicles and oocyte maturation rate in the entire group, age-stratified analysis revealed a negative relationship. Analysis revealed that although traditional treatment determinants such as follicular size and gonadotropin dosing were considered, peak estradiol levels were consistently identified as significant predictors of treatment outcomes.

**Conclusions:**

Physicians may modify treatments for fertility preservation, emphasizing a higher gonadotropin dosage to maximize oocyte retrieval. Elevated estradiol levels can serve as a real-time predictive marker for the number of mature oocytes. While treatment strategies can influence outcomes, intrinsic patient factors, particularly baseline ovarian function, remain crucial. These results challenge beliefs regarding the importance of larger follicles and suggest the need for a tailored approach, considering patient age and specific fertility preservation objectives.

## Introduction

In 2012, the American Society for Reproductive Medicine (ASRM) acknowledged that oocyte vitrification for women facing potential fertility decline was no longer experimental (Practice Committees of the ASRM and the Society for Assisted Reproductive Technology 2013). This endorsement, coupled with evolving lifestyle choices, technological advancements, and heightened fertility awareness, has propelled the rise of social fertility preservation in recent years [10]. In 2014, the ASRM provided a fact sheet on their patient education platform, detailing the use of oocyte vitrification for women who are not necessarily confronted with fertility-threatening diseases, a practice commonly referred to as social fertility preservation [3]. This form of assisted reproductive treatment attracts a demographic that is markedly different from traditional patient populations—it primarily includes healthy women with no known fertility disorders [17].

In many countries, even in those who fund IVF treatments, public health insurance does not cover these treatments, placing the financial burden on the patients [4, 6, 16]. This financial aspect influences treatment goals, with an emphasis on maximizing the number of oocytes retrieved in the shortest possible time, while minimizing patient expenses.

Distinct challenges and considerations arise in the field of social fertility preservation. The types and dosages of medications prescribed for these patients may vary compared to other IVF cohorts. Some physicians might recommend higher dosages or prolonged follicular stimulation periods to maximize oocyte retrieval. It is essential to consider how these potential treatment variations influence the overall outcomes.

This study sought to determine whether treatment approaches for fertility preservation patients, especially concerning timing of oocyte retrieval, differ from those given to other IVF patients and to evaluate the rationale behind any observed differences.

## Materials and methods

This retrospective cohort study was conducted at a tertiary, university-affiliated medical center. It was approved by the Meir Medical Center Ethics Committee (Reference number: MMC-0393–20). The study included 571 patients who completed an assisted reproductive treatment and underwent oocyte retrieval from February 2012 to October 2022. Of these, 165 were undergoing fertility preservation, and 406 served as controls. The control group was women who underwent IVF treatment due to male factor indication without known infertility problems of their own. Case–control matching was implemented based on patient age and baseline FSH levels. After matching, each group included 140 patients.

Inclusion criteria were patients ages 30–41 years, with no medical conditions and a normal ovarian reserve. Patients who were determined to have poor ovarian reserve according to the guidelines set by the Israeli Ministry of Health (AFC < 7, FSH > 10, AMH < 25%) for their age [1, 2] were excluded. The study group consisted of patients undergoing social fertility preservation only.

All patients were treated with an antagonist protocol, with ovarian stimulation initiated on day 2 of the menstrual cycle.

The initial gonadotropin dose was based on a comprehensive assessment of the patient’s age, baseline ovarian reserve markers, and other clinical factors. After stimulation was initiated, estradiol levels were monitored to assess follicular development, and the gonadotropin dosage was adjusted accordingly. Trigger injections were administered when at least two leading follicles reached 17 mm or larger, and estradiol levels were adequate. Gonadotropin dosage and timing of triggering were determined by the physician.

For ovulation triggering, either GnRH-agonist 0.2 mg decapeptyl (0.1 mg, 105 µg triptorelin acetate, Ferring, Germany) or r-hCG 250 mcg (Ovitrelle, 250 µg choriogonadotropin alfa, Merck Serono S.A., Switzerland) was administered. Decapeptyl was primarily used in fertility preservation cycles, while the choice of triggering medication in the control group was based on patient characteristics and risk for ovarian hyperstimulation syndrome (OHSS).

Oocyte retrieval was performed 36 h after triggering. Primary outcomes were number of oocytes retrieved and mature oocyte rate. Secondary outcomes were recovery rate (RR) and the rate of large follicles (ROLF).

The mature oocyte rate was calculated as the number of MII oocytes divided by the total number of retrieved oocytes per cycle. We defined a cut-off of 80% as a good oocyte maturation rate ( ESHRE Special Interest Group of Embryology and Alpha Scientists in Reproductive Medicine. 2017). This cut-off will be referred to as the optimal maturation rate in this study. The RR was defined as the number of oocytes retrieved divided by the number of follicles present on the day of triggering. The RR was defined according to the study of Bosdou et al. [7] who calculated it as the number of oocytes divided by the number of 11 mm follicles. They reported a mean RR of 62.5%, which was suitable for our unit’s retrieval methods. To evaluate the significance of follicle size on the day of triggering in relation to outcomes, we introduced a parameter termed ROLF. The ROLF is derived from the ratio of the number of follicles measuring greater than 17 mm to the anticipated total number of follicles exceeding 10 mm in diameter (as seen on ultrasonographic examination on triggering day). The rationale behind establishing this parameter was to ascertain whether the proportion of larger follicles, which are often considered more mature and potentially more viable for fertilization, is directly correlated with successful IVF outcomes. By utilizing ROLF, we aimed to provide a standardized measure that can offer insights into the optimal range of follicle sizes for achieving favorable IVF outcomes.

The RR was defined according to the study of Bosdou et al. [7] who calculated it as the number of oocytes divided by the number of 11-mm follicles. They reported a mean RR of 62.5%, which was suitable to our unit’s retrieval methods.

### Statistical methods

Data were analyzed using SPSS, version 28.0 (IBM Corp., Armonk, NY, USA). All statistical tests were two-tailed, and a *p*-value less than 0.05 was considered statistically significant.

Continuous variables were assessed for normality using visual inspection of histograms and the Q-Q plot. Since some variables did not follow a normal distribution, results were reported as medians with interquartile ranges (25th and 75th percentiles). Differences between two independent groups were evaluated using the Mann–Whitney *U* test. Categorical variables were compared between groups using the chi-square test.

The elective fertility preservation and control groups were matched based on age (± 1 year) and FSH serum levels (± 0.5 IU/l). The matched groups were compared using the Wilcoxon signed-rank test for continuous variables and the McNemar test for categorical variables.

Spearman’s rank correlation coefficient was used to assess the relationships between continuous variables such as patient characteristics, treatment parameters, and IVF outcomes.

The chi-square automatic interaction detector method was applied to identify the best predictor for > 10 mature oocytes [13]. Variables including age, BMI, baseline values, number of stimulation and antagonist days, starting and total dose of gonadotrophins, triggering medication, and blood estradiol levels on triggering day were considered potential predictors.

## Results

A total of 571 patients were initially included in the study, with 165 patients undergoing social fertility preservation treatment and the 406 patients in the control group receiving treatment due to male factor indication. Matching resulted in 140 patients in each group. The demographic parameters before matching are presented in Table 1.

**Table 1:** Baseline characteristics of the study groups before matching

Variable	Elective oocyte preservation (*n* = 165)	Control (*n* = 406)	*p*-value
Age (years)	35.8 (34.2–37.1)	34.4 (32.1–37.4)	< 0.01
BMI (kg/m^2^)	23.0 (20.0–26.0)	25.0 (21.0–29.0)	< 0.01
Baseline FSH (IU)	7.7 (6.6–9.1)	7.1 (5.9–8.8)	< 0.01

Data are presented as median (interquartile range), *BMI*, body mass index

Treatment characteristics are presented in Table 2. No significant difference was observed between women undergoing social fertility preservation and control patients in terms of duration of stimulation (10.0 days [9.0–11.0] vs. 9.0 days [8.0–11.0], *p* = 0.21). The choice of gonadotropin medication was also similar. Most patients undergoing social fertility preservation were triggered with GnRH-agonist (97.9%), while 92.1% of the control group were triggered with Ovitrelle. The initial gonadotropin dose was higher in the social fertility preservation group than in controls (300.0 IU [225.0–300.0] vs. 225.0 IU [150.0–243.0], *p* < 0.01). Estradiol levels were higher in the study group compared to controls (2635.0 pg/ml [1714.0–3592.0] vs. 1289.5 pg/ml [911.0–1787.5], *p* < 0.01).

**Table 2:** Treatment parameters

Treatment characteristics	Elective oocyte preservation (*n* = 140)	Control (*n* = 140)	*p*-value
Duration of stimulation, days (range)	10.0 (9.0–11.0)	9.0 (8.0–11.0)	0.21
Starting dose, IU (range)	300.0 (225.0–300.0)	225.0 (150.0–243.0)	< 0.01
Total gonadotropin dose, IU (range)	2712.0 (2250–3300)	2025.0 (1610–2925.0)	< 0.01
Triggering medication, % (*n*)			
Ovitrelle	2.1 (3)	92.1 (129)	< 0.01
Decapeptyl	97.9(137)	7.9 (11)	
HMG/recombinant			
Menopur % (*n*)	72.4 (21)	89.7% (26)	0.18
Pergoveris % (*n*)	27.6 (8)	10.3 (3)	
FSH/LH + FSH			
FSH, % (*n*)	14.3 (20)	12.1 (17)	0.73
FSH + LH, % (*n*)	85.7 (120)	87.9 (123)	
Duration of antagonist, days (range)	5.0 (4.0–6.0)	4.0 (3.0–5.0)	< 0.01
E2 on ovulation triggering day, pg/ml (range)	2635.0 (1714.0–3592.0)	1289.5 (911.0–1787.5)	< 0.01
LH on ovulation triggering day, IU/L (range)	1.9 (1.0–3.1)	1.3 (0.8–2.5)	< 0.01
Progesterone on ovulation triggering day, nmol/L (range)	0.8 (0.5–1.0)	0.6 (0.4–0.9)	** < 0.01**

Data are presented as median (interquartile range) or % (*n*)

Follicular characteristics according to sonographic examination on the day of triggering are presented in Table 3. More follicles of any size were found in the study compared to the control group (*p* < 0.01). The ROLF was similar between groups (34.1% [21.1–50.0] in social fertility preservation vs. 36.4% [20.0–50.0] in the control group, *p* = 0.51).

**Table 3:** Characteristics of aspirated follicles

Number of follicles according to size	Elective oocyte preservation	Control	*p*-value
> 20 (*n*)	1.0 (0.0–1.0)	0.0 (0.0–1.0)	< 0.01
19–20 (*n*)	1.0 (0.0–2.0)	1.0 (0.0–1.0)	< 0.01
17–18 (*n*)	2.0 (1.0–3.0)	1.0 (1.0–2.0)	< 0.01
15–16 (*n*)	2.0 (1.0–4.0)	1.0 (1.0–2.7)	< 0.01
10–14 (*n*)	5.0 (3.0–10.0)	3.0 (2.0–5.0)	< 0.01
Total follicles expected	13.0 (9.0–19.0)	7.0 (4.2–10.0)	< 0.01
Rate of large follicles (%)*	34.1 (21.1–50.0)	36.4 (20.0–50.0)	0.51

Data are presented as median (interquartile range). *Rate of large follicles was calculated as the number of follicles > 17 mm divided by the total expected number of follicles > 10 mm

A comparison of treatment outcomes is presented in Table 4. More oocytes were retrieved in the fertility preservation group compared to the control group (12.0 [7.0–17.0] vs. 7.0 [3.0–11.0], *p* < 0.01). There was no significant difference in the oocyte maturation rate between the social fertility preservation and control groups (79.3 [66.7–89.5] vs. 75.9 [56.7–100.0], respectively, *p* = 0.29). The RR was also comparable between the groups (92.0 [66.7–116.4] vs. 91.7 [66.7–124.4], respectively, *p* = 0.69).

**Table 4:** Comparison of IVF outcomes between groups

Variable	Elective oocyte preservation	Control group	*p*-value
Number of retrieved oocytes	12.0 (7.0–17.0)	7.0 (3.0–11.0)	< 0.01
Number of mature oocytes	10.0 (5.0–13.7)	5.0 (2.0–8.0)	< 0.01
Maturation rate (%)	79.3 (66.7–89.5)	75.9 (56.7–100)	0.29
Recovery rate (%)	92.0 (66.7–116.4)	91.7 (66.7–124.4)	0.69

Data are presented as median (interquartile range). Recovery rate was calculated as the number of oocytes retrieved during retrieval divided by the number of follicles present on the day of triggering

Data were then analyzed for fertility preservation treatments to assess the correlations between treatment characteristics and outcomes. There was no correlation between patient age and ROLF (*r* = 0.03, *p* = 0.70). A negative correlation was observed between ROLF and the number of oocytes retrieved for the entire group (*r* =  − 0.40, *p* < 0.01), as well as according to patients’ age of > 36 (*r* =  − 0.46, *p* < 0.01) compared to ≤ 36 (*r* = 0.13, *p* < 0.01). There was no correlation between ROLF and maturation rate for the entire group (*r* = 0.06, *p* = 0.47), but interestingly, when analyzed according to age ≤ 36 or > 36, there was a negative correlation (*r* =  − 0.37, *p* < 0.01; *r* =  − 0.43, *p* < 0.01). Maturation rate was not correlated with duration of stimulation (*r* = 0.004, *p* = 0.96). ROLF and recovery rate were not correlated for the entire group (*r* = 0.08, *p* = 0.32), or according to patients’ age ≤ 36 (*r* =  − 0.14, *p* = 0.20) or > 36 (*r* = 0.01, *p* = 0.91).

**Fig. 1:**
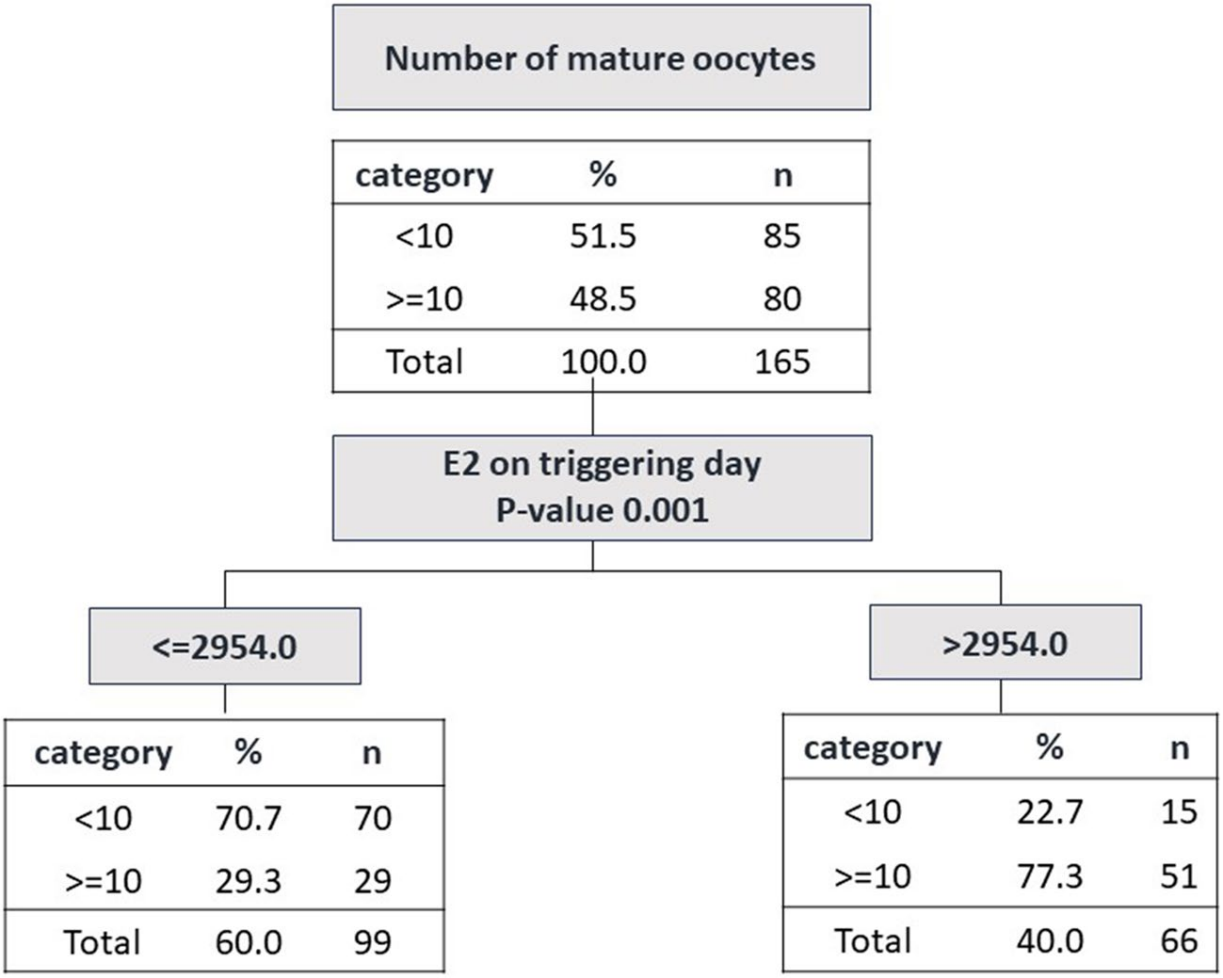
Tree diagram to identify the best predictor for > 11 mature oocytes

In the tree diagram used to predict the ideal number of mature oocytes, we found that estradiol level on triggering day was the main predictor variable (Fig. 1). When serum estradiol was > 2954 pg/ml, 77% of the patients had more than 10 mature oocytes and when it was ≤ 2954 pg/ml, 29.3% had more than 10 mature oocytes. When evaluating the ROLF in fertility preservation cycles based on the optimal RR value, categorized as either ≤ 62% or > 63%, no significant difference was observed (43.6% [23.2–56.7] vs. 34.8% [20.0–50.0], *p* = 0.25). Similarly, there was no significant difference in the ROLF when comparing cycles according to the optimal maturation rate, with values of 35.3% [20.0–50.0] for a maturation rate of ≤ 80% and 35.7% [21.7–54.0] for a maturation rate of > 80% (*p* = 0.51).

## Discussion

The findings presented here highlight distinct treatment approaches for fertility preservation patients compared to a control group with similar characteristics. The rising prevalence of fertility preservation patients in the IVF landscape, especially in regions like Israel where IVF treatments are fully covered by medical insurance, highlights the importance of these findings. It is crucial to note that while IVF treatments are publicly funded in Israel, social fertility preservation treatments are not, which requires patients to pay for it themselves. This financial distinction was a primary motivator for this study, prompting us to explore how it might impact the course and outcomes of treatments. We selected a control group of women undergoing IVF due to male factors, hypothesizing that their likely normal fertility potential would offer a comparable reference point to the social fertility preservation group. Additionally, we aimed for the treatments in the control group to be composed primarily of intracytoplasmic sperm injection (ICSI), as the removal of cumulus cells is crucial for observing the mature oocyte—a process comparable to oocyte freezing.

A key observation of this study is a higher gonadotropin starting dose administered with more retrieved oocytes in the fertility preservation group compared to the control group. This suggests that physicians may approach the treatment of fertility preservation patients differently, modifying their treatment plans to suit this specific group of women. Additionally, the increased number of oocytes retrieved in the fertility preservation group might be a direct result of the higher gonadotropin dosage.

The present study supports the findings of another report on women undergoing two successive IVF cycle attempts for elective egg freezing. The results indicate that increasing the daily gonadotropin dose above 300 IU resulted in a higher yield of mature oocytes [15]. This further emphasizes the potential benefits of adjusting the gonadotropin dosage in specific scenarios. However, other research challenges this approach. A randomized controlled trial by Van Tilborg et al. focused on the efficacy of individualized FSH dosing based on the ovarian reserves of women starting IVF/ICSI. They found that for patients with a regular menstrual cycle, a standard FSH starting dose of 150 IU per day yielded results comparable to those of an individualized FSH dose [18]. The primary outcomes revolved around the effects of individualized dosing based on birth rates and OHSS incidence. While their study reported similar live birth rates across the group, the individualized FSH dosing led to fewer oocytes retrieved in the first treatment cycle [18]. Although that study primarily included infertile patients and utilized a GnRH-agonist protocol in about 80% of the cycles, the present study focused on a different demographic. Considering these differences, one can postulate that the gonadotropin dose has a key function in fertility preservation cycles.

Notably, in our study, none of the women was hospitalized due to OHSS. This suggests that in the context of fertility preservation, the risk of OHSS is decreased, particularly due to the widespread use of GnRH-agonist triggering. which is known to significantly reduce the risk of this complication [8]. This allows for greater flexibility and potentially higher dosages of gonadotropins without the same level of concern for OHSS, as is seen with hCG triggering.

While individualized dosing may optimize certain outcomes, as seen in the study by Van Tilborg et al., the specific requirements of fertility preservation seem to require a tailored approach to gonadotropin dosing to maximize oocyte retrieval, unconstrained by the usual concerns regarding OHSS.

However, the observation of Van Tilborg et al. regarding live birth rates with a fixed dose of 150 IU highlights the need for further exploration. Given the differences in study foci and outcomes, further research is needed to examine the live birth rates in the context of different dosing strategies.

Interestingly, despite the differences in oocyte yield, the ROLF remained comparable between groups. This suggests that while the overall number of oocytes retrieved might differ, the proportion of larger follicles remains consistent. At the outset of this study, we hypothesized that the fertility preservation group would undergo a longer duration of stimulation, leading to larger follicles and a distinct ROLF compared to the control group. However, our findings indicate that even with different treatment approaches between the groups, the duration of stimulation was consistent, which did not translate into a significant difference in ROLF.

Upon closer examination of the fertility preservation cohort alone, additional insights regarding follicle size and oocyte retrieval were observed. Interestingly, a negative correlation was observed between ROLF and the number of oocytes retrieved across the entire group and when evaluated according to age. Similarly, the maturation rate was negatively correlated with ROLF when assessed based on age. A previous study investigating the developmental potential of extremely small follicles demonstrated that small follicles in comparison to large follicles resulted in significantly lower recovery maturation and fertilization rates [5]. While one might intuitively assume that a higher ROLF, indicative of a larger proportion of mature follicles, would lead to a greater overall yield of oocytes, our findings challenge this notion. It appears that larger follicles do not always translate to optimal success in retrieving oocytes. A strategy commonly employed in IVF treatments involves initiating the trigger when several follicles achieve diameters of 17 to 18 mm [9, 14], a protocol our findings seem to align with. In contrast, Yang et al. [19] highlighted the significance of follicle size and quantity, particularly for patients younger than 35 years of age. For this demographic, triggering at a point where large or medium follicles are dominant has been associated with superior oocyte quality. Conversely, for patients ages 35 and older, optimal outcomes have been observed when the proportion of medium follicles is no less than that of small follicles [19]. In the present study, results regarding younger patients differed from those of Yang et al., with trends indicating that a higher proportion of large follicles correlated with less favorable outcomes. Notably, when our analysis incorporated the standard of an optimal maturation rate of 80%, the influence of ROLF on outcomes became less noticeable. While traditional beliefs and some studies emphasize the importance of larger follicles for oocyte retrieval and quality, our findings emphasize the need to reevaluate these assumptions, especially considering age-specific nuances and the evolving understanding of follicle development.

The association between estradiol levels and the number of oocytes retrieved is well-established in the scientific literature [12]. Thus, it is not surprising that our study also found a link between elevated estradiol levels and more mature oocytes. This information is crucial as it enables clinicians to predict the outcome of treatment in real time, setting expectations for both the medical team and the patient. Moreover, the predictive power of estradiol levels provides a real-time insight that can be more informative than other treatment parameters. Our model demonstrates that other parameters, which are often emphasized in clinical practice, such as follicular size, duration of stimulation, and starting dose of gonadotropins, may not influence outcomes as strongly as is sometimes thought. It seems that a patient’s baseline ovarian function, which is represented by peak estradiol levels during treatment, might have a more significant role in determining outcomes. This notion emphasizes the importance of individualizing patient care and the need to include ovarian reserve markers when devising treatment plans. It underscores the notion that while treatment modifications can influence outcomes, intrinsic patient factors remain paramount.

One of the primary strengths of this study is its thorough case–control matching based on age and baseline FSH levels, which aimed to ensure a balanced comparison between the fertility preservation and control groups. Our inclusion criteria, focusing on patients 30–41 years of age with no medical conditions and normal ovarian reserves, allowed for a homogeneous cohort, reducing potential confounding variables that could affect results. Furthermore, this research is novel in its exploration of whether physicians tailor their treatment approaches differently for fertility preservation patients compared to other groups. This analysis is relevant given the increasing prevalence of fertility preservation and the potential implications of treatment decisions on outcomes. By highlighting these distinctions and their potential effects, the present study provides a foundation for future investigations.

This study had several limitations. Its retrospective nature inherently carries potential biases, including those of selection and information. The cohort, which was obtained from a single tertiary center, may not be entirely representative of broader populations, thereby potentially limiting the generalizability of the findings. While the control group was matched for its expected similarity to the fertility preservation group, it is essential to acknowledge that those patients were undergoing fertility treatment due to failed pregnancy attempts. Consequently, some may have unexplained infertility in addition to a male factor. This might have influenced their treatment parameters and outcomes, suggesting that the smaller number of oocytes observed could be attributed to these potential underlying issues rather than the increased gonadotropin dosages alone.

An additional limitation of this study is the reliance on FSH levels and patient age for matching. While FSH levels were included in the standard assessment for ovarian function throughout the study period, we recognize the potential advantages of incorporating AMH levels as a more informative marker of ovarian reserve. Unfortunately, due to the retrospective nature of the study and the lack of comprehensive AMH data for all participants, we could not employ AMH for matching purposes. Future prospective studies should consider using AMH as a matching parameter to potentially enhance the accuracy and relevance of case–control comparisons in fertility research.

Another limitation relates to the difference in triggering agents used in the groups. The majority of fertility preservation patients were triggered with GnRH-agonist, while most of the control group received r-hCG triggering. Therefore, this disparity could have introduced variability in the outcomes observed. For instance, the higher number of oocytes retrieved in the fertility preservation group might not solely be attributed to the higher gonadotropin dosages but could also be influenced by the triggering agent’s effect on final oocyte maturation. A previous study found that the outcomes of GnRH-agonist triggering were comparable to those of r-hCG triggering in patients undergoing social preservation cycles [11]. Future studies that control for the type of triggering agent used would provide more definitive insights into the relative impacts of gonadotropin dosages and triggering agents on IVF outcomes.

An additional limitation is the focus on oocyte number and maturation as primary outcomes. While these parameters are crucial for understanding the immediate impact of fertility preservation strategies, the ultimate goal is successful pregnancies. Thus, our conclusions, while illuminating, will not be definitive until we ascertain the outcomes when these frozen oocytes are used in subsequent IVF cycles.

A final point is that given the evolving landscape of IVF treatments, our findings might be influenced by changes in clinical practices over the decade-long span of the study. Future prospective studies with larger, diverse cohorts and controlled parameters will further refine our understanding of fertility preservation strategies.

## Conclusions

This study illuminates the intricate relationship between gonadotropin dosing, follicle size, and oocyte retrieval in fertility preservation. The findings suggest that postponing triggering may not necessarily improve IVF outcomes, and challenge traditional beliefs favoring larger follicles. A nuanced approach, considering patient age and specific fertility preservation objectives, is essential.

## Data Availability

Data will be made available to the editors of the journal for review or query upon request.

## References

[1] Almog B, Shehata F, Suissa S, Holzer H, Shalom-Paz E, La Marca A, Muttukrishna S, Blazar A, Hackett R, Nelson SM. Age-related normograms of serum antimüllerian hormone levels in a population of infertile women: a multicenter study. Fertil Steril. 2011;95(2359–2363):e1. 21457958 10.1016/j.fertnstert.2011.02.057

[2] Ash N. Expansion of the health services package for 2022. In: Circular DGS (Ed.): Israeli Ministry of Health. 2022.

[3] ASRM Patient Education Committee. Can i freeze my eggs to use later if i'm not sick? : https://www.reproductivefacts.org/news-and-publications/fact-sheets-and-infographics/can-i-freeze-my-eggs-to-use-later-if-im-not-sick/. 2014.

[4] De Proost M, Coene G, Nekkebroeck J, Provoost V. ‘I feel that injustice is being done to me’: a qualitative study of women’s viewpoints on the (lack of) reimbursement for social egg freezing. BMC Med Ethics. 2022;23:35. 35351108 10.1186/s12910-022-00774-z PMC8966350

[5] Avraham S, Kalma Y, Azem F, Zakar L, Amir H, Rahav R, Barzilay L, Dviri M, Almog B. Morphokinetic characteristics of embryos originating from extremely small follicles: a prospective study. Euro J Obstet Gynecol Reprod Biol. 2019;238:110–3. 10.1016/j.ejogrb.2019.05.017 31128533

[6] Baldwin K. Egg freezing, fertility and reproductive choice: negotiating responsibility, hope and modern motherhood Emerald Publishing Limited. 2019.

[7] Bosdou JK, Kolibianakis EM, Venetis CA, Zepiridis L, Chatzimeletiou K, Makedos A, Triantafyllidis S, Masouridou S, Mitsoli A, Tarlatzis B. Is the time interval between HCG administration and oocyte retrieval associated with oocyte retrieval rate? Reprod Biomed Online. 2015;31:625–32. 26387934 10.1016/j.rbmo.2015.08.005

[8] Engmann L, Diluigi A, Schmidt D, Nulsen J, Maier D, Benadiva C. The use of gonadotropin-releasing hormone (GNRH) agonist to induce oocyte maturation after cotreatment with GNRH antagonist in high-risk patients undergoing in vitro fertilization prevents the risk of ovarian hyperstimulation syndrome: a prospective randomized controlled study. Fertil Steril. 2008;89:84–91. 10.1016/j.fertnstert.2007.02.002. 17462639 10.1016/j.fertnstert.2007.02.002

[9] Fauque P, Lehert P, Lamotte M, Bettahar-Lebugle K, Bailly A, Diligent C, Clédat M, Pierrot P, Guénédal M-L, Sagot P. Clinical success of intrauterine insemination cycles is affected by the sperm preparation time. Fertil Steril. 2014;101(1618–1623):e3. 24745729 10.1016/j.fertnstert.2014.03.015

[10] Gil-Arribas E, Blockeel C, Pennings G, Nekkebroeck J, Velasco JAG, Serna J, De Vos M. Oocyte vitrification for elective fertility preservation: a swot analysis. Reprod Biomed Online. 2022;44:1005–14. 35304091 10.1016/j.rbmo.2022.02.001

[11] Herzberger EH, Knaneh S, Amir H, Reches A, Ben-Yosef D, Kalma Y, Azem F, Samara N. Gonadotropin-releasing hormone agonist versus recombinant human chorionic gonadotropin triggering in fertility preservation cycles. Reprod Sci. 2021;28:3390–6. 10.1007/s43032-021-00622-2. 34076872 10.1007/s43032-021-00622-2

[12] Hughes EG, Robertson DM, Handelsman DJ, Hayward S, Healy DL, De Kretser DM. Inhibin and estradiol responses to ovarian hyperstimultion: effects of age and predictive value for in vitro fertilization outcome. J Clin Endocrinol Metab. 1990;70:358–64. 2298853 10.1210/jcem-70-2-358

[13] Kass GV. An exploratory technique for investigating large quantities of categorical data. J Roy Stat Soc: Ser C (Appl Stat). 1980;29:119–27.

[14] Loumaye E, Engrand P, Shoham Z, Hillier SG, Baird DT. Clinical evidence for an LH ‘ceiling’effect induced by administration of recombinant human LH during the late follicular phase of stimulated cycles in World Health Organization type i and type ii anovulation. Hum Reprod. 2003;18:314–22. 12571167 10.1093/humrep/deg066

[15] Orvieto R, Aizer A, Saar-Ryss B, Marom-Haham L, Noach-Hirsh M, Haas J, Nahum R. Elective egg freezing patients may benefit from increasing the maximal daily gonadotropin dose above 300iu. Reprod Biol Endocrinol. 2022;20:1–6. 36536380 10.1186/s12958-022-01049-3 PMC9762009

[16] Shenfield F, De Mouzon J, Scaravelli G, Kupka M, Ferraretti A, Prados F, Goossens V. The ESHRE working group on oocyte cryopreservation in Europe. Oocyte and ovarian tissue cryopreservation in european countries: Statutory background, practice, storage and use. Hum Reprod Open. 2017;1:1–9. 10.1093/hropen/hox003 PMC6276651 30895222

[17] Van Loendersloot LL, Moolenaar LM, Mol BWJ, Repping S, Van Der Veen F, Goddijn M. Expanding reproductive lifespan: a cost-effectiveness study on oocyte freezing. Hum Reprod. 2011;26:3054–60. 21896545 10.1093/humrep/der284

[18] Van Tilborg TC, Oudshoorn SC, Eijkemans MJ, Mochtar MH, Van Golde RJ, Hoek A, Kuchenbecker WK, Fleischer K, De Bruin JP, Groen H. Individualized FSH dosing based on ovarian reserve testing in women starting IVF/ICSI: a multicentre trial and cost-effectiveness analysis. Hum Reprod. 2017;32:2485–95. 29121350 10.1093/humrep/dex321

[19] Yang J, Gao J, Wang Y, Liu H, Lian X. Impact of follicular size categories on oocyte quality at trigger day in young and advanced-age patients undergoing GNRH-ant therapy. Front Endocrinol. 2023;14:1167395. 10.3389/fendo.2023.1167395 PMC10140496 37124736

